# Study of starch aging characteristics based on Terahertz technology

**DOI:** 10.1002/fsn3.2417

**Published:** 2021-06-28

**Authors:** Tao Wang, Shuya Wang, Chen Zhai, Liang Wang, Yunfeng Xie, Qian Li, Xu Zheng

**Affiliations:** ^1^ College of Life Science and Technology Xinjiang University Xinjiang China; ^2^ Nutrition and Health Research Institute COFCO Corporation Beijing Key Laboratory of Nutrition and Health and Food Safety Beijing China; ^3^ China Communication Technology (Jiang Men) Corporation Guangdong China; ^4^ Shenzhen Institute of Terahertz Technology and Innovation Guangdong China

**Keywords:** crystallinity, days, regeneration degree, starch aging, terahertz

## Abstract

Traditional methods for the determination of starch aging indicators often have a series of shortcomings such as time‐consuming, high cost, large human error, damage to samples, environmental pollution, and high requirements for inspectors. Therefore, it is meaningful to find or establish a dynamic fingerprint identification pattern that can detect the aging degree of starch during the process of processing or storage quickly and accurately. It not only provides guidance for starch food processing but also saves a lot of human, material resources, and time. Terahertz technology is an emerging molecular spectroscopy technology in the 21st century. It is with low energy and basically harmless to the human body. It can also realize nondestructive testing of samples. In the experiment, the samples were prepared by the tableting method and the samples containing 20% of 50 mg samples were prepared with polyethylene as the diluent. The thickness of the samples was 1 mm and the diameter was 13 mm. The terahertz time‐domain spectrometer was used to obtain the spectral information of aging starch at different aging times. After the pretreatment of the spectrum by vector normalization, first derivative, and multiple scattering correction, the prediction models of aging days, crystallinity, and resilience of aging starch were established, respectively. The determination coefficient (*R* ^2^) of the established models is all greater than 95%, indicating that the established models are highly reliable and can be used to predict the aging days, crystallinity, and retrogradation degree of starch. And the *R* ^2^ of the prediction model based on the refractive index spectrum is greater than that of the absorption coefficient spectrum. The experimental method obtains the dynamic fingerprint identification map of starch in the aging process, realizes the real‐time monitoring and detection of the starch aging process, and provides an effective means for the production and processing of starch‐related industries.

## Graphical abstract

The determination coefficients (*R* ^2^) of the established models were all greater than 95%, indicating that the established models had high reliability and could be used to predict the days, crystallinity, and degree of regression of starch aging. This paper provides a solid foundation for real‐time monitoring and determination of aged starch.

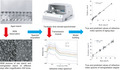

## 1 INTRODUCTION

The phenomenon that the starch milk turns into a paste after cooking, baking, and other heating processes is called starch gelatinization (Li et al., 2019). For fully gelatinized starch, when the temperature drops to a certain level, the system is in a thermodynamically non‐equilibrium state due to insufficient molecular thermal energy (Funami et al., 2005; Jiang et al., 2020). The molecular chains are attracted and arranged by hydrogen bonds to reduce the free enthalpy of the system (Eliasson & Gudmundsson, 1996). Starch molecules and water molecules are matched and rearranged in space conformation to achieve an orderly stable state of system equilibrium (Chang et al., 2004; Chen, Chen, et al., 2018; Teo et al., 2000). At this time, the straight parts of amylose and amylopectin tend to be arranged in parallel, returning from the amorphous form to the crystalline form to produce a hardening phenomenon. This process is called starch aging or retrogradation (Van Soest & Knooren, 1997; Vodovotz & Chinachoti, 1998). Starch will age during processing, transportation, and storage (Bertoft, 2017; Yazid et al., 2018). Aging starch products not only have poor sensory quality but also greatly reduce their nutritional value (Mahmood et al., 2017). This is due to the increase in hydrogen bonding between adjacent molecules, forming a microcrystalline bundle structure, which is not easy to be digested by amylase (Chen et al., 2015; Krystyjan et al., 2013). However, in some food processing processes, it is necessary to add appropriate retrograded starch to enhance the taste and certain physiological functions, such as vermicelli and noodles. Therefore, it is meaningful to understand the process of starch aging. However, conventional methods for the determination of physical and chemical indexes of aged starch mostly adopt classical chemical methods, which have the disadvantages of long time, high cost, and great human error. Terahertz (THz) technology is a new detection technology, which can realize rapid quantitative analysis of substances by using predictive models.

Terahertz waves are electromagnetic waves with frequencies from 0.1 to 10 THz (Siegel, 2002). Terahertz spectrum generates terahertz electric field through the interaction between the terahertz pulse and the sample. The waveforms in the time domain and the reference frequency domain are obtained through the fast Fourier transform, and then the waveforms in the frequency domain are processed (Hou et al., 2021; Liu & Fan, 2020). The absorption coefficient, refractive index, and dielectric constant of the sample can be extracted. According to these parameters, the quantitative detection of substance can be realized (Siegel et al., 2004; Zeitler et al., 2007). The vibration frequencies of many organic molecules are located in the middle infrared frequency band, but the intermolecular forces such as hydrogen bond, low‐frequency vibration of lattice, and macromolecular skeleton vibration all occur in the terahertz band. These vibrations reflect the molecular structure information, to determine the components contained in the material and the classification of molecular configuration (Dragoman & Dragoman, 2004; Kemp et al., 2003).

Nakajima et al. (2019) determined the starch content during the germination of mung bean by Terahertz spectroscopy. Guan et al. used the terahertz time‐domain spectroscopy to analyze the alum ratio in sweet potato starch qualitatively and quantitatively (Guan & Chao, 2019). Terao et al. (2018) have studied the boson peak dynamics of natural polymer starch using terahertz time‐domain spectroscopy and low‐frequency Raman scattering. Liu et al. have studied the potassium aluminum sulfate dodecahydrate in potato starch by terahertz spectroscopy qualitatively and quantitatively (Chen, 2013; Liu et al., 2020). Chen, Singh, et al. (2018)) used terahertz time‐domain spectroscopy to measure glucose levels in the blood of diabetic patients quantitatively. It can be seen from the above studies that the terahertz spectrum can also be used to achieve qualitative and quantitative analysis of different substances.

Intermolecular forces such as hydrogen bonds, low‐frequency vibrations of the crystal lattice, and macromolecular skeleton vibrations all occur in the terahertz band, and these vibrations all reflect molecular structure information. The aging process of starch is the hardening phenomenon produced by the continuous association of hydrogen bonds between molecular chains. Therefore, it is feasible to use terahertz technology to study the relevant characteristics of starch aging. In this study, a terahertz time‐domain spectrometer was used to collect the terahertz spectrum at different aging times from 0 to 35 days. After preprocessing the spectra by vector normalization, first derivative and other preprocessing methods, the models of aging days, crystallinity, and retrogradation were established. The determination coefficients (*R* ^2^) of the established models were all greater than 95%, showing that the established models had high reliability and could be used to predict the days, crystallinity, and degree of regression of starch aging. This method saves a lot of manpower and material resources for the determination of starch‐related properties and provides a solid foundation for future research on starch‐related properties.

## 2 MATERIALS AND METHODS

### 2.1 Materials and reagent

Wheat starch; polyethylene; alpha‐amylase; acetate buffer solution (pH = 5.6); NaOH solution; HCl solution; I_2_‐KI solution.

### 2.2 Equipment

Terahertz time‐domain spectrometer (CCT‐1800); Milli‐Q ultra‐pure water system (Milli‐Q Reference); Powder press machine (BJ‐15); X‐ray diffractometer (Bruker D8); UV‐visible spectrophotometer (U‐3900); pH meter (PB‐10).

### 2.3 Methods

#### 2.3.1 Preparation of aged starch

Add 20 g starch into 200 ml deionized water, mix it well, and put it into a sealed sterilization pot. Heat it under steam at 120℃ for 20 min to gelatinize it. The gelatinized samples were quickly cooled to room temperature and stored in a refrigerator at 4℃ for 0, 1, 3, 5, 7, 14, 21, 28, and 35 days. After taking it out, freeze, dry, and crush it at −80℃ and pass through 100‐mesh sieve. Keep in a dryer for later determination.

#### 2.3.2 Scanning electron microscopic observation of raw starch and retrograded samples

The SU‐3500 scanning electron microscope of Japan HITACHI company was used for micro‐scanning. Place the raw starch and each retrograded sample evenly on the carbon conductive glue and fix it on the sample table and then observe. Observe with different magnifications and angles such as 50×, 100×, 200×, 400×, 800×, 1,200×, 2,000×, etc. Use the software that comes with the instrument to take pictures.

#### 2.3.3 Determination of physical and chemical indexes of aged starch

##### Determination of crystallinity of aged starch

XRD technique measured the crystallinity of aged starch. After the starch is completely gelatinized, there is no endothermic peak in XRD. When starch ages, internal amylose, and amylopectin are rearranged to form recrystallization. At this time, the crystal structure of starch is destroyed and additional energy must be added. Therefore, the endothermic peak will appear in the aged starch in XRD, and the greater the degree of starch aging, the greater the endothermic peak. Therefore, the crystallinity of aged starch can be measured by XRD technology (Doumeng et al., 2020; Hu et al., 2020).

The specific determination method is as follows: The Bruker D8 XRD instrument was used for analysis, using monochromatic Cu‐Kα rays with a wavelength of 0.1542 nm. The starch sample powder balanced overnight in a water environment was laid out in a groove on the sample table and a slide was used to ensure a smooth surface. The test conditions were as follows: the tube pressure is 40 kV and the tube flow is 40 mA. The scanning area is 4–50° and the step width is .02°. The scanning mode was continuous and the number of repeats was 1. The area calculated by integrating under the XRD diffraction peak is the crystallinity of the aged starch.

##### Determination of retrogradation degree of aged starch

The longer the retrogradation degree time was the greater the resistance of starch to iodine chromogenic reaction was (Abd Karim et al., 2000). This experiment adopts the alpha‐amylase method that was developed for the determination of starch retrogradation degree, the specific method is accurate according to samples from 25 mg of starch aging, adding ultra‐pure water 8 ml, and shock blender. Then add 3.5 u/ml α‐amylase solution 2 ml and 0.1 mol/L acetate buffer solution (pH 5.6) 2 ml, after incubating at 37℃ for 10 min, add 4 mol/L NaOH solution 5 ml to stop the enzyme reaction, and then use 4 mol/L HCl to adjust the pH of the solution to medium The final volume is adjusted to 100 ml. Take out 10 ml of the hydrolyzed solution, add 5 ml of 0.2% I_2_‐2% KI solution, and dilute to 100 ml again. After standing for 20 min, measure its absorbance at 625 nm with an ultraviolet‐visible spectrophotometer (Tsuge et al., 1990). The formula for calculating the degree of regeneration as follows:

(1) $$D=\frac{a‐b}{a‐c}\times 100\%$$

where *a* is the absorbance of native starch, *b* the absorbance experimental starch and *c* the absorbance of completely gelatinized starch.

#### 2.3.4 Acquisition and modeling of Terahertz spectrum of aged starch

There are two forms of terahertz spectrum, one is reflection spectrum, which is suitable for substances with strong and weak absorption of terahertz wave, and the other is transmission spectrum, which is suitable for substances with moderate absorption of Terahertz wave. The transmission spectrum has certain requirements on the thickness of the sample. If the sample is too thick or the water content is too large, too much Terahertz radiation will be absorbed, the sample signal waveform will be completely submerged in background noise, or the difference between amplitude and noise at the peak point in time domain is too small, resulting in too low signal‐to‐noise ratio. If the sample is too thin or the moisture content is too low, the difference between the sample signal waveform and the reference signal is small, and the change of the material cannot be reflected obviously.

The instrument used in this experiment recommended the best sample mass fractions of 5%, 10%, and 20%. Considering the characteristics of the sample thickness and the difficulty in forming by adding polyethylene pressed sheet, this experiment adopted the pressed sheet with the sample mass fraction of 20%, the total mass of 50 mg, the thickness of about 1 mm, and the diameter of 13 mm. The aged starch samples were placed in the terahertz time‐domain spectrometer to collect information such as time domain, frequency domain, refractive index, and absorption coefficient. Combining preprocessing methods such as the first derivative and vector normalization to preprocess the spectrum. The processed spectrum was used to model and predict the days, crystallinity, and retrogradation degree of aged starch.

## 3 RESULTS AND DISCUSSION

### 3.1 Morphology observation of starch under SEM

**FIGURE 1:**
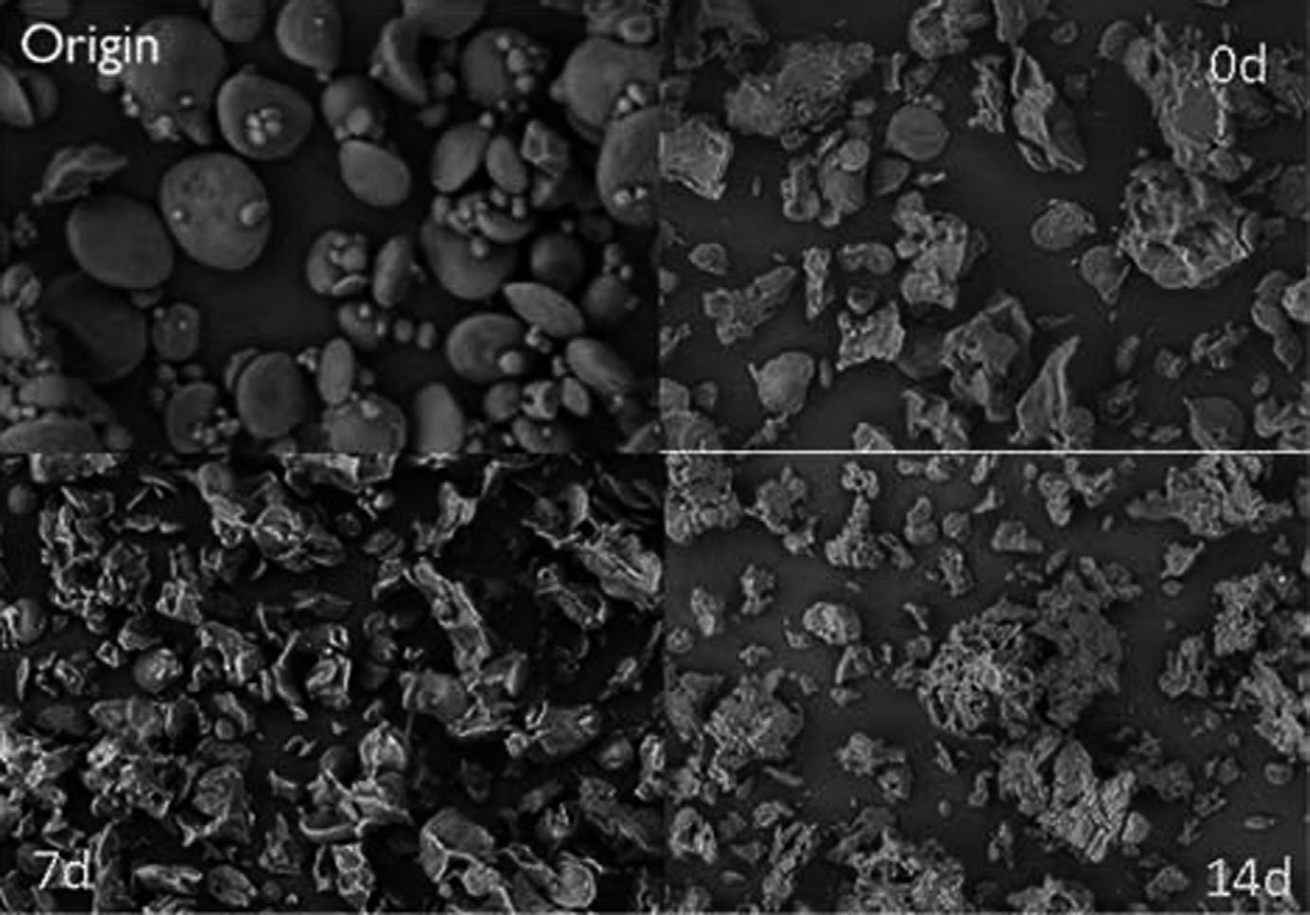
SEM pictures of raw starch and retrograded starch of different days after magnification 200 times

Figure 1 shows the structure of starch granules taken at 200× for native starch and samples with the aging time of 0, 7, and 14 days. The SEM observation of the gelatinized starch in the time period from 0, 7 to 14 days after retrogradation was not obvious. But compared with raw starch, the gelatinized starch granules basically no longer retain the complete granular structure. This observation shows that the sample is gelatinized more thoroughly and can be used as the analysis sample.

### 3.2 Analysis of physical and chemical indexes of aged starch

**FIGURE 2:**
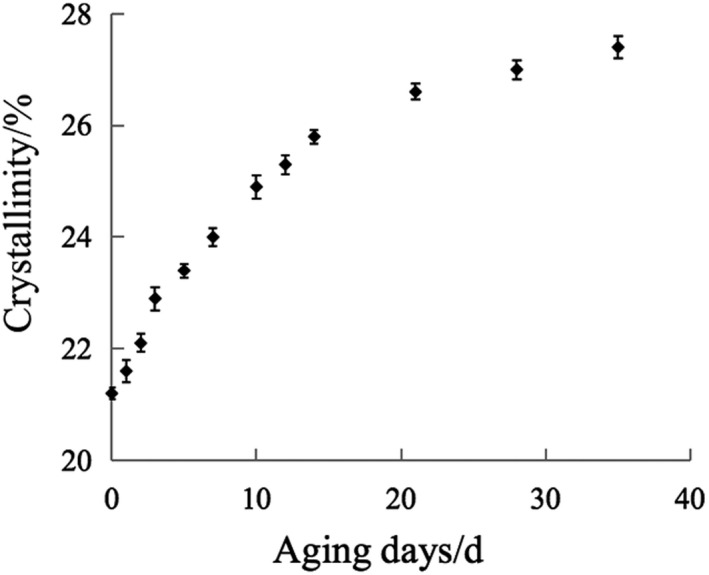
The curve of crystallinity of aged starch with time

**FIGURE 3:**
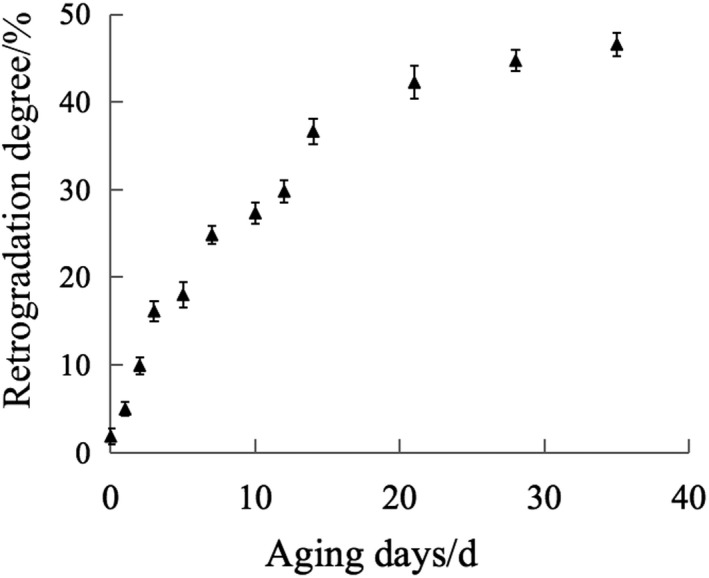
The curve of retrogradation degree of aged starch with time

The crystallinity and retrogradation degree of aged starch measured by XRD and α‐amylase method were analyzed and the results are in Figures 2 and 3. [Correction added on 19 July 2021, after first online publication: The Figures 2 and 3 has been relocated under the Section 3.2.]

For fully gelatinized starch, when the temperature drops to a certain level, the system is in a thermodynamically non‐equilibrium state due to insufficient molecular thermal energy. The molecular chains are attracted and arranged by hydrogen bonds to reduce the free enthalpy of the system. Starch molecules and water molecules are matched and rearranged in space conformation to achieve a stable and orderly arrangement of system equilibrium (Dong et al., 2020). At this time, the straight parts of amylose and amylopectin tend to be arranged in parallel, returning from an amorphous form to a crystalline form (Zheng et al., 2020). The longer the retrogradation time of starch, the greater the resistance to iodine chromogenic reaction was. Therefore, during the aging process of starch, the degree of crystallinity and retrogradation gradually increase with time. This is consistent with the experimental results. Zou used the α‐amylase method to determine the retrogradation of aged starch and used near‐infrared and mid‐infrared technology to model and predict the retrogradation of starch (Zou et al., 2018). The experimental results are consistent with the results of this study. It shows that the established prediction model has a certain degree of persuasiveness.

### 3.3 Terahertz spectra of aged starch

**FIGURE 4:**
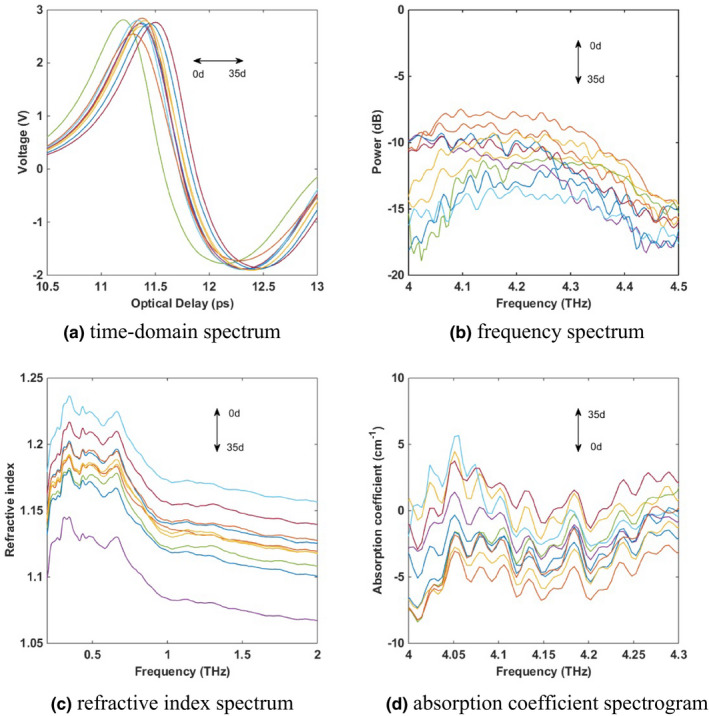
Terahertz spectra of aged starch

The starch samples aged for 0, 1, 2, 3, 5, 7, 12, 14, 21, and 35 days were selected for spectral collection, as shown in Figure 4.

Since the sample in this experiment is solid and the spectrum is acquired by transmission method, the refractive index spectrum and absorption coefficient spectrum can effectively reflect the characteristic changes of starch during the aging process. Figure 4a is the time‐domain spectrum, Figure 4b is the frequency‐domain spectrum, Figure 4c is the refractive index spectrum and Figure 4d is the absorption coefficient spectrum. The spectrum of frequency domain is obtained by Fourier transform from the time‐domain spectrogram. Collect the reference time‐domain signal *E*_ref_ (*t*) when the sample is not placed and the sample time‐domain signal *E*_sam_ (*t*) when the sample is placed. The frequency‐domain signals *E*_ref_ (*ω*) and *E*_sam_ (*ω*) of the reference and sample are obtained after Fourier transform. The refractive index and absorption coefficient of the sample were obtained by data processing using the methods proposed by Dorney and Duvillaret (Dorney et al., 2001; Duvillaret et al., 1999). The formulas are as follows.

The refractive index was calculated according to the following equation:

(2) $$n\left(\omega \right)=\frac{\varphi \left(\omega \right)\;c}{\omega \;d}+1$$

where *n*(*ω*) is the refractive index of the sample and *ω* the frequency. *φ*(*ω*) the phase difference between the sample and the reference signal, $\varphi \left(\omega \right)={\varphi \;\;}_{\mathrm{sample}}\left(\omega \right)‐{\varphi \;}_{\mathrm{ref}}\left(\omega \right)$. where *c* is the speed of light and *d* the thickness of the sample.

We can obtain the absorption coefficient from the following equation:

(3) $$\alpha \left(\omega \right)=\frac{2}{d}In\left\{\frac{4n\left(\omega \right)}{A\left(\omega \right){\left[n\left(\omega \right)+1\right]}^{2}}\right\}$$

where *α*(*ω*) is the absorption coefficient of the sample and *n*(*ω*) the refractive index of the sample. *A*(*ω*) is the amplitude ratio of the sample to the reference signal. Where *d* is the thickness of the sample and ω is the frequency.

The time‐domain spectrum, frequency‐domain spectrum, refractive index spectrum, and absorption coefficient spectrum of aging starch are inherently related. With the prolonging of aging time, the crystallinity increased gradually, the content of regenerated starch increased and the absorption of terahertz wave was enhanced. As a result, the transmittance of the terahertz wave is weakened, the time required for complete transmittance is increased and the peak intensity is reduced with a time delay phenomenon. In Figure 4, the time‐domain spectrogram shows a delayed phenomenon when the aging time gradually increases. The refractive index in the refractive index spectrum gradually decreases when the aging time gradually increases and the absorption coefficient in the absorption coefficient spectrum gradually increases when the aging time gradually increases. This is because, the recovered starch content increases with the extension of aging time. The experimental results are consistent with the theory and the trend is stable, which provides a good basis for this study to establish a prediction model of aged starch by using terahertz spectroscopy technology.

### 3.4 The establishment of Terahertz prediction model

#### 3.4.1 Terahertz technology detects starch aging days

Analyze the obtained spectra, preprocess the spectra firstly and find the optimal preprocessing method by preprocessing the spectra through different preprocessing methods. Table 1 shows the results of various pretreatment methods.

**TABLE 1:** Pretreatment optimization results of aging days

Spectrum	Number	Pretreatment method	RMSECV	*R* ^2^	Dimension	RPD
Refractive index	1	The second derivative	1.01	99.09	10	10.5
	2	First derivative	2.05	96.25	10	5.17
	3	First derivative+MSC	2.24	95.51	10	4.72
	4	First derivative+SNV	2.27	95.4	10	4.66
	5	Vector normalization (SNV)	2.91	92.43	10	3.63
Absorption coefficient	6	The second derivative	1.04	98.77	10	8.93
	7	Multiple scattering correction	1.54	97.26	10	6.08
	8	First derivative +SNV	2.82	90.81	10	3.3
	9	Vector normalization (SNV)	3.03	89.42	10	3.07
	10	Multiple scattering correction	3.19	88.22	10	3.19

**FIGURE 5:**
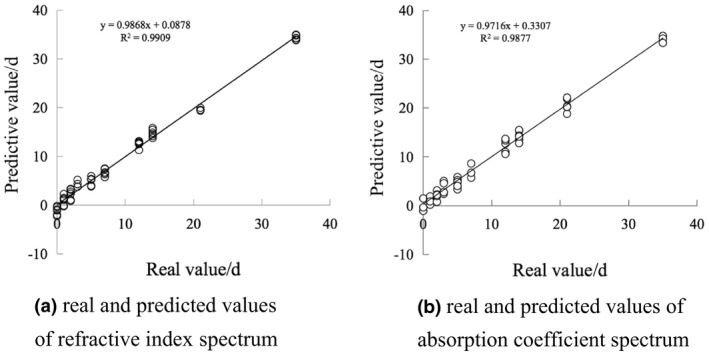
Model diagram of predicted aging days and real aging days under optimal conditions

The higher the determination coefficient *R* ^2^ is, the more consistent the analysis results are with the chemical analysis results, and the higher the reliability is. The lower the RMSECV is, the closer the predicted value obtained during cross‐validation in the calibration modeling process is to the chemical analysis value and the higher the accuracy of the calibration model is. It can be seen from the table that the pretreatment method of the optimal model of refractive index spectrum is the second derivative. In the optimal model, *R* ^2^ is 99.09 and RPD is 10.5. The preprocessing method of the optimal absorption coefficient spectral model is the second derivative. In the optimal model, *R* ^2^ is 98.77 and RPD is 8.93. The optimal model is selected as the model for predicting starch aging days. Figure 5 shows the relationship between the true value and the predicted value under this model. Figure 5a is the relationship between the true value and the predicted value of the refractive index spectrum. Figure 5b is the relationship between the true value of the absorption coefficient spectrum and the predicted value. It shows that the model has a good fit and can be used as a model to predict the age of starch.

#### 3.4.2 Terahertz technology to detect starch crystallinity

Table 2 shows the results of various pretreatment methods, and then optimizes the spectrum, and selects the best model as the model for predicting starch aging crystallinity.

**TABLE 2:** Pretreatment optimization results of crystallization

Spectrum	Number	Pretreatment method	RMSECV	*R* ^2^	Dimension	RPD
Refractive index	1	The second derivative	.0914	99.87	10	28.3
	2	Multiple scattering correction	.314	98.52	10	8.21
	3	First derivative	.318	98.47	10	8.1
	4	First derivative+SNV	.328	98.38	10	7.87
	5	First derivative+MSC	.331	98.35	10	7.79
Absorption coefficient	6	First derivative	.515	95.79	10	4.87
	7	Eliminate constant offsets	.515	95.77	9	4.88
	8	First derivative +MSC	.553	95.14	10	4.53
	9	Vector normalization (SNV)	.602	94.22	10	4.16
	10	Eliminate constant offsets	.553	93.07	8	3.82

**FIGURE 6:**
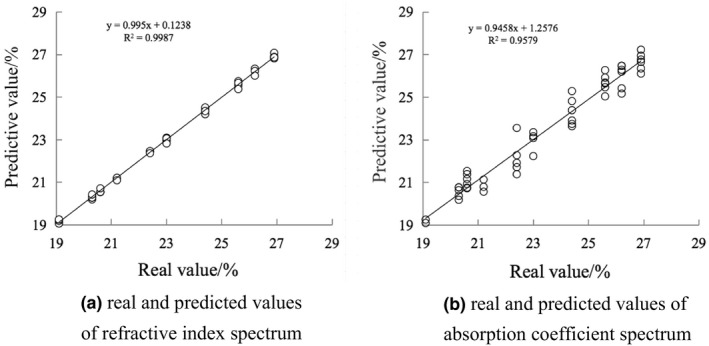
Model diagram of predicted and true values of crystallinity under optimal conditions

It can be seen from Table 2 that the preprocessing method of the optimal model of the refractive index spectrum is the second derivative. In the optimal model, *R* ^2^ is 99.87 and RPD is 28.3. The preprocessing method of the optimal model of absorption coefficient spectrum is the first derivative. In the optimal model, *R* ^2^ is 95.78 and RPD is 4.87. The dimension of the model is as shown in Figure 6. Figure 6a is the relationship between the true value and the predicted value of the time‐domain spectrogram, and Figure 6b is the relationship between the true value and the predicted value of the frequency‐domain spectrogram. This shows that the model has a good fitting degree and can be used as a model to predict the crystallinity of starch aging.

#### 3.4.3 Terahertz technology to detect starch retrogradation degree

Table 3 shows the results of various pretreatment methods, and then optimizes the spectrum, and selects the best model as the model for predicting starch aging crystallinity.

**TABLE 3:** Pretreatment optimization results of retrogradation degree

Spectrum	Number	Pretreatment method	RMSECV	*R* ^2^	Dimension	RPD
Refractive index	1	The second derivative	2.08	98	10	7
	2	First derivative+MSC	4.17	91.77	10	3.49
	3	First derivative+SNV	4.46	90.83	10	3.3
	4	Multiple scattering correction	4.72	90.44	8	3.23
	5	Vector normalization (SNV)	6.05	83.65	9	2.47
Absorption coefficient	6	The second derivative	3.15	95.34	9	4.63
	7	First derivative+SNV	4.09	89.66	10	3.11
	8	First derivative	4.87	85.32	9	2.61
	9	Multiple scattering correction	4.55	84.97	5	2.58
	10	Vector normalization (SNV)	5.3	82.83	9	2.41

**FIGURE 7:**
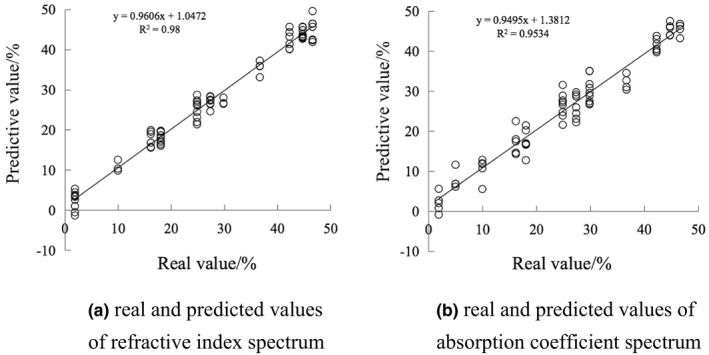
Model diagram of predicted and true values of retrogradation degree under optimal conditions

It can be seen from Table 3 that the preprocessing method of the optimal model of the refractive index spectrum is the second derivative. In the optimal model, *R* ^2^ is 98 and RPD is 7. The preprocessing method of the optimal model of the absorption coefficient spectrum is the second derivative. In the optimal model, *R* ^2^ is 95.34 and RPD is 4.63. The dimension of the model is, as shown in Figure 7. Figure 7a is the relationship between the true value and the predicted value of the time‐domain spectrogram, and Figure 7b is the relationship between the true value and the predicted value of the frequency‐domain spectrogram. It shows that the model has a good fit and can be used as a model for predicting starch aging retrogradation degree.

## 4 CONCLUSION

The aging phenomenon of starch in the process of processing, transportation, and storage will seriously affect the sensory and quality of starch food. Therefore, it is meaningful to understand the aging process of starch. The study on the crystallinity, and retrogradation degree of aged starch changes with aging time show that with the extension of aging time, the crystallinity, and retrogradation degree of aged starch increase. The pretreatment methods used in the optimal prediction model of the refractive index (*R* ^2^ = 99.04) and absorption coefficient spectra (*R* ^2^ = 98.74) of aging days are both the second derivative. The preprocessing methods used in the optimal prediction model of the refractive index (*R* ^2^ = 99.87) and absorption coefficient spectra (*R* ^2^ = 95.87) of crystallinity are the second derivative and the first derivative. The preprocessing methods used in the optimal prediction model of the refractive index (*R* ^2^ = 98) and absorption coefficient spectra (*R* ^2^ = 95.34) of retrogradation degree are both the second derivative. In this study, the terahertz time‐domain spectrometer was used to model and predict the aging days, crystallinity, and regenerative degree of starch with *R* ^2^ greater than 95%, indicating that the terahertz time‐domain spectrometer can be used for qualitative and quantitative detection of bioactive substances and other macromolecular substances. Through comparison, it can be seen that the *R* ^2^ of the prediction model based on the refractive index spectrum is greater than that of the absorption coefficient spectrum. Therefore, the model based on refractive index spectrum has little deviation in actual production. The experimental method obtains the dynamic fingerprint identification map of starch in the aging process, realizes real‐time monitoring and detection of the starch aging process, and provides an effective means for the production and processing of starch‐related industries.

## CONFLICT OF INTEREST

The authors declare that there are no conflicts to declare.

## AUTHOR CONTRIBUTIONS

Tao Wang: Data curation (lead); Investigation (lead); Methodology (lead); Software (lead); Writing‐original draft (lead); Writing‐review & editing (lead). Shuya Wang: Conceptualization (equal); Resources (equal). Chen Zhai: Conceptualization (equal); Funding acquisition (lead); Project administration (lead); Resources (lead). Liang Wang: Data curation (equal); Resources (equal). Yunfeng Xie: Funding acquisition (equal); Resources (equal). Qian Li: Data curation (equal); Software (lead). Xu Zheng: Data curation (equal); Software (equal).

## Data Availability

The data that support the findings of this study are available from the corresponding author upon reasonable request.
